# Transitions in depression: if, how, and when depressive symptoms return during and after discontinuing antidepressants

**DOI:** 10.1007/s11136-022-03301-0

**Published:** 2022-11-23

**Authors:** Arnout C. Smit, Evelien Snippe, Laura F. Bringmann, H. J. Rogier Hoenders, Marieke Wichers

**Affiliations:** 1 ICPE, Department of Psychiatry, University of Groningen, University Medical Center Groningen, Triade Building Entrance 24, HPC CC72, 9700 VB Groningen, The Netherlands; 2 Faculty of Behavioral and Movement Sciences, Clinical Psychology, VU Amsterdam, Amsterdam, The Netherlands; 3 Department of Psychometrics and Statistics, Faculty of Behavioural and Social Sciences, University of Groningen, Groningen, The Netherlands; 4 Center for Integrative Psychiatry, Lentis Mental Health Institution, Groningen, The Netherlands

**Keywords:** Depressive disorder, Antidepressant discontinuation, Ecological Momentary Assessment, Personalized psychiatry, Meaningful change

## Abstract

**Purpose:**

The aim of the current study is to provide insight into *if*, *how,* and *when* meaningful changes occur in individual patients who discontinue antidepressant medication. Agreement between macro-level quantitative symptom data, qualitative ratings, and micro-level Ecological Momentary Assessments is examined.

**Methods:**

During and shortly after antidepressant discontinuation, depressive symptoms and ‘feeling down’ were measured in 56 participants, using the SCL-90 depression subscale weekly (macro-level) for 6 months, and 5 Ecological Momentary Assessments daily (micro-level) for 4 months (30.404 quantitative measurements in total). Qualitative information was also obtained, providing additional information to verify that changes were clinically meaningful.

**Results:**

At the macro-level, an increase in depressive symptoms was found in 58.9% of participants that (a) was statistically reliable, (b) persisted for 3 weeks and/or required intervention, and (c) was clinically meaningful to patients. Of these increases, 30.3% happened suddenly, 42.4% gradually, and for 27.3% criteria were inconclusive. Quantitative and qualitative criteria showed a very high agreement (Cohen’s *κ* = 0.85) regarding if a participant experienced a recurrence of depression, but a moderate agreement (Cohen’s *κ* = 0.49) regarding how that change occurred. At the micro-level, 41.1% of participants experienced only sudden increases in depressed mood, 12.5% only gradual, 30.4% experienced both types of increase, and 16.1% neither.

**Conclusion:**

Meaningful change is common in patients discontinuing antidepressants, and there is substantial heterogeneity in how and when these changes occur. Depressive symptom change at the macro-level is not the same as depressive symptom change at the micro-level.

Reaching remission has long been the main concern in the treatment of depression. Unfortunately, even if remission is reached, it is likely that symptoms will return [1, 2], especially when antidepressants are discontinued [3, 4]. Preventing meaningful increases in depressive symptoms is an essential step toward improving long-term quality of life in patients with depression. Though a lot of research has focused on identifying risk factors [1, 2, 5], and preventing recurrence through antidepressant maintenance treatment or preventive therapies [4, 6, 7], surprisingly little research has focused on the actual process through which depressive symptoms return.

To investigate thoroughly the processes during which depressive symptoms recur in individuals, a shift in methodology is required. The process of symptom development occurs within-person, over time. To study such processes Nelson et al. [8] suggested three research designs that would be highly valuable. First, macro-level longitudinal assessment (i.e., repeated assessment over an extended time period, ideally covering the entire period in which symptoms change), would be helpful in uncovering global patterns of symptom recurrence over time. Secondly, qualitative methods (i.e., first-person accounts of how depressive recurrence was experienced), should be considered. This would be a major addition as it provides valuable information on how quantitative conclusions correspond with what patients experience in the real world. Thirdly, micro-level longitudinal assessment, i.e., high-frequency assessment of fluctuations in depressed mood in daily life (e.g., several times a day), allows the researcher to characterize individual processes of change in the course of daily life [9] and may provide insight into processes that are so subtle or noisy that patients themselves may be unaware of them. The current study (named TRANSitions In Depression: TRANS-ID Tapering) was designed to capture the entire process of depressive symptom recurrence prospectively in patients in remission who tapered their antidepressant medication. By combining all three modes of data collection suggested by Nelson et al. [8], the present study aims to provide new insights in if, how, and when depressive symptoms recur.

To determine *if* a clinically meaningful increase in depressive symptoms occurred, it is important to examine whether a substantial change occurred by assessing quantitatively whether the increase in symptom can be distinguished from measurement error. Though many studies aim to do this by comparing pre-intervention and post-intervention measurements of symptoms [10–12], limitations of such pre-post designs are known and it has been suggested that more frequent measurement would be useful [8, 13, 14]. Another advantage compared to existing studies with a pre-post design is that frequent retesting also allows us to test if the increase in symptoms is persistent, as short-lived changes may represent normal fluctuations in the life of a patient which should not be interpreted as meaningful changes.

Though such quantitative symptom data are important to test if a change in symptoms was quantitatively substantial, they do not show whether a change had a meaningful impact on the daily life of a patient. If a quantitative increase in symptoms did not cause the patient to experience discomfort or if the patient was not even aware of the quantitative change, it is questionable whether that change was truly meaningful. To increase ecological validity, interviews and self-report qualitative data could be rated by independent raters to examine whether depressive symptom increases meaningfully impacted the daily life of patients. Combining a quantitative and a qualitative approach provides insight into the agreement between quantitative and qualitative criteria for meaningful change. Furthermore, if a change satisfies both quantitative and qualitative criteria, this increases the likelihood that this change represents a true and meaningful change in symptoms [15].

Though a substantial amount of research has focused on *if* meaningful change occurs and how to define meaningful change [12, 16, 17], far less attention has been paid to *how* and *when* meaningful change occurs. An important distinction in *how* changes happen is whether the change happens gradually or suddenly. Gradual recurrence implies that depressive symptoms increase little-by-little over a period of time. In the case of a sudden recurrence, symptoms would increase substantially in short timeframes, following a seemingly symptomless period. For a long time, it has been argued that changes in psychiatry should not be viewed exclusively as gradual and linear [13], and sudden improvements in depressive symptoms have been studied extensively [18, 19]. Unfortunately, far fewer studies have investigated sudden development of depressive symptoms, all using retrospective questionnaires to determine the duration of change [20–23]. Regarding *when* changes occur, it is well known that retrospectively studying the timing of events can lead to biased and inaccurate results [24]. The resulting inability to accurately estimate when change occurs has been identified as a substantial limitation of existing studies using a pre-post design [8, 13, 14]. A study with far more frequent assessment may improve understanding of *when* depressive symptoms recur; during, shortly after, or long after discontinuation of antidepressants.

A novel approach to examine meaningful change is to use a method that increases ecological validity and reducing recall bias: Ecological Momentary Assessment (EMA) [25, 26]. EMA research provides a highly detailed, micro-level view of how patients feel in daily life as depressed mood can be assessed multiple times a day. Even though it has been argued that emotional patterns in daily life represent micro-level building blocks of depression that seem to play an important role in the course of mental (ill-)health [27, 28], EMA data have not yet been used to assess meaningful change. By using EMA data, we could gain insight into change that is meaningful in the daily life of patients. Furthermore, such fine-grained prospective data of depressed mood in daily life could enable to establish the timing of change more precisely than macro-level assessments [9]. As current methods for assessing meaningful change in EMA data are limited, statistical methods for examining if, how, and when changes occur in EMA data are needed.

The prospective, intensive, and multimodal design of the present study provides a unique opportunity to investigate methods to examine *if* a meaningful increase in depressive symptoms occurred after remission and to provide insight into *how* and *when* these increases happened. We aim to examine whether quantitative changes in macro-level depressive symptoms correspond with qualitative reports of patients’ experiences of depressive symptoms. We expect that in some cases, quantitative and qualitative criteria for *if* and *how* changes occurred will not match. In addition, we will examine *if*, *how*, and *when* changes in down mood occur using micro-level EMA data. Furthermore, we aim to examine if meaningful increases in depressive symptoms and increases in EMA data of depressed mood happen gradually or suddenly. Finally, we aim to compare the results of the different methods assessing symptom change. As the current study was the first to utilize all three modes of longitudinal data collection suggested by Nelson et al. [8], we also aim to investigate the potential usefulness of combining these modes of data collection when investigating the meaningful change.

## Methods

### Sample

**Fig. 1:**
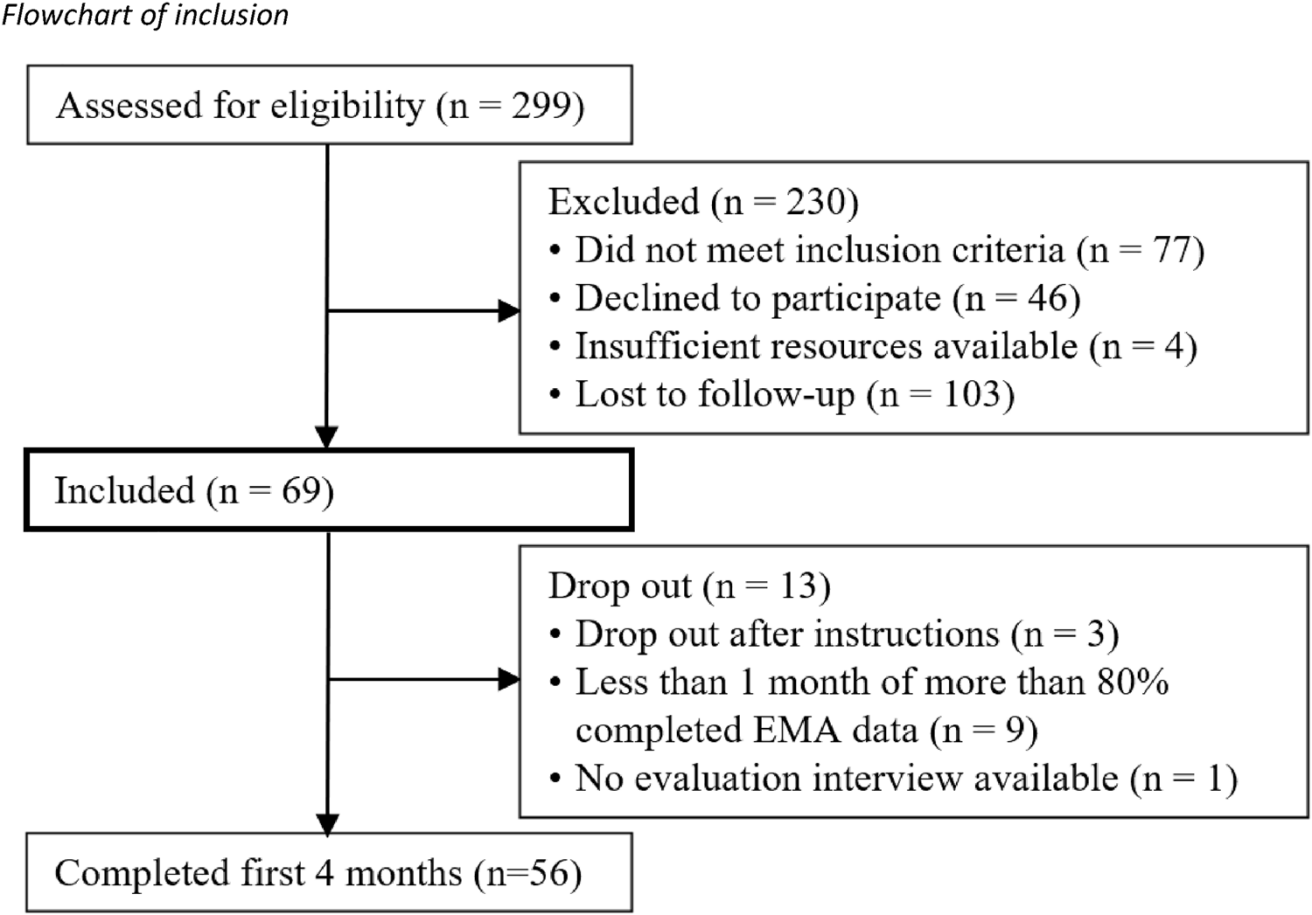
Flowchart of inclusion

Sixty-nine participants were recruited through flyers, advertisement in traditional and social media, several healthcare institutions and a Dutch pharmacy (the ‘Regenboog’ pharmacy), who supplies patients with so-called tapering strips that allowed these participants to reduce their dose using tiny decrements [29]. Participants (a) were on antidepressant medication because of a past depressive episode, (b) were in remission at baseline, and (c) made a shared decision with their general practitioner or psychiatrist to (gradually) stop using medication because of remission. Exclusion criteria were age < 18, current psychotic or manic episode, and reported diagnosis of a personality disorder. Figure 1 shows a flow chart regarding recruitment and inclusion.

### Design

**Fig. 2:**
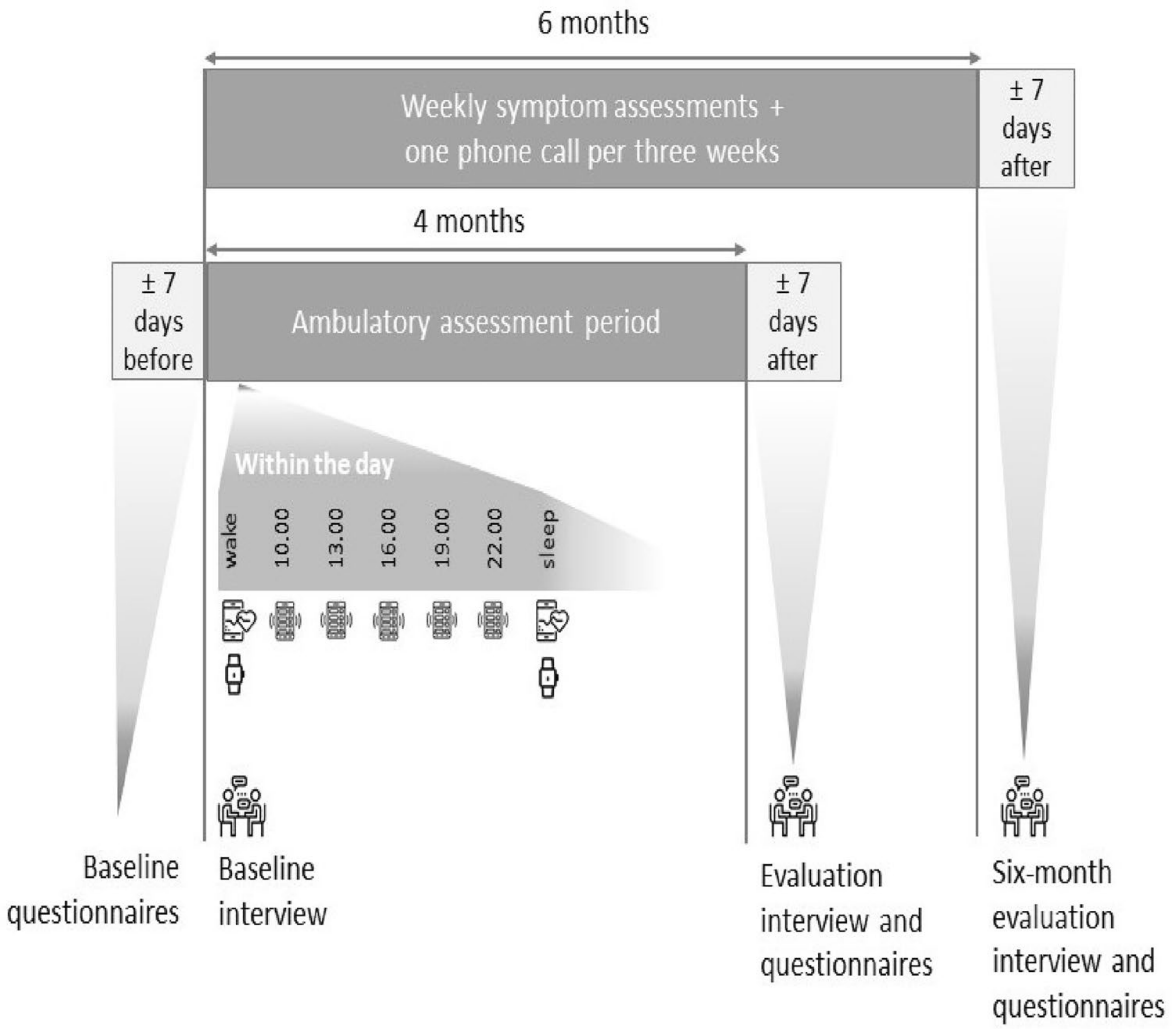
Overview of the participation trajectory: duration of the various assessment periods, timing of the interviews and questionnaires, and the within-day structure for the ambulatory assessments. *Note* Within each day of the ambulatory assessment period, participants completed five 2-min EMA questionnaires at 3-h intervals (the times in the figure are an example). Upon waking, and before sleep, participants measured their heart rate for 5 min. Participants wore an actigraph continuously

The current study is an observational study, where intensive longitudinal assessment was started about a month before the intended date that discontinuation of antidepressants would be completed and covered a period of 6 consecutive months. This allowed us to capture the final steps of antidepressant discontinuation, as well as the first months directly after discontinuation, in which depressive symptoms are most likely to return [30]. Figure 2 shows the study timeline and provides an overview of all assessments. An extended version of the methods including complete descriptions of the baseline questionnaire, actigraphy, heart rate monitoring, and evaluation questionnaires is available on the TRANS-ID Tapering OSF page [31].

### Procedure

#### Inclusion and instructions

Past depressive episode, absence of a current depressive episode (inclusion criteria), psychotic, and/or manic episode (exclusion criteria) were assessed using the mini-SCAN, a validated semi-structured interview designed to systematically assess DSM-IV criteria for psychiatric disorders [32].

If all criteria were met, participants were given a detailed instruction for the ambulatory assessments. During this instruction, participants practiced filling out the ambulatory questionnaires on their own smartphone. Researchers followed a detailed protocol to give concrete examples and reactions to a participant’s response style. Participants received a leaflet repeating the most important instructions. In two follow-up phone calls (~ 2 days and ~ 1 week after the baseline interview), the key points of the instructions were repeated, and any questions that had emerged were answered. The full instructions are available in Dutch upon request.

If EMA completion rate dropped below 80%, participants were called to discuss and resolve any issues or concerns that were preventing them from filling out the questionnaire. Planned phone calls were used to obtain additional qualitative information (~ 1 call every 3 weeks; 6 months).

### Materials

#### Baseline questionnaire

Demographics were obtained in an online baseline questionnaire which the participants completed in the week before the ambulatory assessment period started.

#### Macro-level assessment

Each weekend for a period of 6 months, participants filled out the Symptom Checklist (SCL)-90 depression subscale [33] on their smartphone (~ 26 assessments per participant). Two items, on suicidal thoughts and self-esteem, were excluded because of the potential burden they may impose on participants when administered at this frequency.

#### Micro-level assessment

The EMA protocol covered a period of 4 months and consisted of 5 measurements a day, which were sent via text-message at fixed intervals of 3 h, with a reminder-text after 10 min and a 30 min window for completion (~ 600 assessments per participant). A measurement took about 2 min to complete and the timings were set according to the sleep–wake habits of the participant. The full EMA questionnaire is available on OSF [31].

For the current study, we only analyzed the item ‘I feel down’ as it was the closest momentary proxy for depressive symptoms, which was the primary focus of this study. This item was assessed on a 0–100 Visual Analogue Scale. The last EMA questionnaire of each day inquired the dosage of antidepressant medication the participant took that day, which was used to construct an accurate view of the tapering schedule.

#### Qualitative information

In both the macro- and micro-level questionnaires described above, each questionnaire ended in a text-field in which participants were instructed to report any remarkable or influential events. Furthermore, the weekly questionnaire contained an open question inquiring if participants felt their depressive symptoms were heading in the wrong direction. During 3-weekly phone calls, the researchers inquired how participants were doing in terms of mood, depressive symptoms, and treatment.

#### Evaluation questionnaires and interviews

When micro-level data collection ended (after 4 months), changes in medication and psychological treatment were inquired in an online evaluation questionnaire.

Both after 4 months (when daily EMA assessments ended) and 6 months (when weekly assessments ended), participants were invited for a semi-structured evaluation interview aimed at obtaining a detailed picture of a participant’s experiences and depressive symptoms over time. Questions included: Compared to the start of the study period, did you notice a change in depressive symptoms (open question), and to what extent? (7-point Likert scale); Would you describe the change in depressive symptoms as sudden or gradual? (both open and 7-point Likert scale); How did tapering of antidepressant medication influence you, and did tapering have a positive or negative effect on your mood? (both open and 7-point Likert scale); Did you deviate from the planned tapering schedule, and if yes, when and why? (open questions). The full evaluation interview is available in Dutch upon request.

### Processing qualitative information

#### Rating

Information from (a) open comments in daily and weekly questionnaires, (b) emails and phone calls, and (c) open questions in the evaluation interviews was used to assess the real-life experience of depressive symptoms qualitatively. Three raters, two psychologists (A.C.S and E.S.) and one psychiatrist (H.J.R.H.), assessed this information.

Raters assessed the following questions: “Did the patient experience a change in depressive complaints that had a clear and meaningful impact on the daily life of the patient” (Yes/No/Unknown), where ‘meaningful’ was defined as the patient clearly noticing the change in daily life and/or experiencing discomfort as a result of the change, “Did the participant describe the increase in their depressive symptoms as sudden?” (Yes/No/Unknown), and “On which date did the participant first report they were suffering from an increase in depressive symptoms?”. Raters used a rating guide describing which materials should be assessed for each question, and how questions should be rated. Raters were instructed to base conclusions primarily on prospective information from comments and phone calls, as retrospective information from the evaluation interviews may contain recall bias. To diminish the probability of biased ratings or important information being overlooked, raters first rated all participants independently, and later discussed all participants until a consensus rating was reached. Qualitative results in this paper are based on this final consensus rating.

### Macro-level

There were two quantitative criteria to determine if a transition happened at the macro-level: criterion 1a, *reliable change*, and criterion 1b, *persistent change*. Using the reliable change index (RCI) [34], the increase (i.e., one-sided) from the average SCL-90 score of the first two weeks (*y*_baseline_) and each of the subsequent SCL-90 measurements (*y*_*t*_) can be considered reliable at *α* = 0.05 if (*y*_*t*_ *−* *y*_baseline_)/sqrt(2*SEM^2^) > 1.645, where SEM was the standard error of the measurement. Rearranging, substituting either the norms for primary healthcare (SEM = 3.55) or general practice patients (SEM = 3.50) [35], and rounding to the nearest possible score yielded *a cut-off of* (*y*_*t*_ *−* y_baseline_) ≥ 8.5. Criterion 1a, *Reliable change*, was satisfied if depressive symptoms were 8.5 or more points higher than the average level of depressive symptoms during first two weeks of the study.

Criterion 1b, *persistent change*, was satisfied if the reliable elevation in SCL-90 score persisted for at least 3 consecutive weeks. Some participants may have restarted medication or treatment within those 3 weeks, which may interfere with this criterion. As medical doctors are not expected to start treatment when they expect the symptoms to resolve rapidly without intervention, we also regarded criterion 1b as satisfied in participants for whom treatment for depressive symptoms was started or increased, or tapering was interrupted.

We checked the agreement between the quantitative symptom change at the macro-level (i.e., both a reliable and persistent change) and the qualitative rating by calculating the Cohen’s *κ*.

If a reliable increase, defined as an increase larger than 8.5 points in the SCL-90 score, occurred *within one week*, the increase was viewed as “quantitatively sudden,” unless the participant indicated they had experienced the transition as very gradual (1), gradual (2), or somewhat gradual (3) on the evaluation question “To what extent was the increase in your depressive symptoms sudden, on a scale from 1 (very gradual) to 7 (very sudden)?”. We checked whether this corresponded with the qualitative of the rating of *how* participants described the increase in depressive symptoms.

At the macro-level, *when* the increase occurred was defined as the week in which depressive symptoms measured using the SCL-90 were reliably higher (defined using the RCI) compared to (a) previous week for the ‘quantitative sudden’ group, and (b) the first two weeks of the study for the ‘quantitative gradual’ group. If the RCI was significant more than once, we used the week that corresponded most closely with the timing determined in the consensus rating of the qualitative information.

### Micro-level

*If*, *how*, and *when* transitions happened at the micro-level were all determined using a statistical method. A continuous trend in ‘I feel down’ over time was fitted using a Gaussian kernel local linear regression with a bandwidth of 35 observations. We extended this method to test the significance and timing of both sudden and gradual changes in the data.

At each datapoint i, a left-sided Gaussian kernel local linear estimate of the level of ‘I feel down’ was subtracted from a right-sided estimate.^1^ This quantity is expected to be large at times when a sudden increase occurred [36]. At each local extremum, significance of sudden changes was tested using a bootstrap procedure similar to the procedure developed by Gijbels and Goderniaux [37], to determine *if* and *when* significant sudden changes in depressive symptoms occurred.

A gradual trend was fitted in each segment using the Gaussian kernel local linear regression, treating significant sudden changes as additional end-points. A bootstrap procedure was used to estimate where in these gradual segments the slope was significantly larger than 0, estimating *if* and *when* significant gradual increases in depressive symptoms occurred.

As this technique had not been used to analyze the EMA data before and no guidelines existed for choosing the α-level, an α of 0.01 was used in the current study, as this seemed to reduce the probability of a gradual increase being classified incorrectly as a sudden increase in pilot data (see [38]) compared to using the more standard *α* = 0.05. An extensive description, walkthrough, and annotated R code of this method can be found on OSF [39].

## Results

Participants completed median of 27 weekly assessments of depressive symptoms, and a median of 542.5 EMA self-reports (i.e., respectively, a median of 100% and 87.6% compliance), yielding a total of 30.404 completed questionnaires in total. Age ranged from 23 to 71 (mean = 45.9, SD = 12.9), and the sample included 9 males (16.1%). Table 1 shows the information on antidepressants and tapering in the sample.

**Table 1:** Antidepressant medication tapered or discontinued in the sample

Type of medication	Frequency	Dose at baseline Mean (SD)	Percentage using tapering strips (%)	Tapered to 0 mg and did not restart
Venlafaxine	17	17.5 (10.7)	94.1	76.5%^a^
Paroxetine	11	4.3 (2.8)	90.9	63.6%^a^
Sertraline	8	24.6 (20.7)	75.0	87.5%
Fluoxetine	6	4.8 (2.1)	100	83.3%
Citalopram	9	7.9 (4.6)	55.6	100%
Escitalopram	2	3.8 (*)	0	*
Clomipramine	1	*	0	*
Quetiapine	1	*	0	*
Bupropion	1	*	0	*

^a^In both of these groups, one participant completely discontinued venlafaxine or paroxetine, but did start on a different antidepressant due to an increase in depressive symptoms. These were *not* counted as participants who tapered to 0 mg and did not restart. When these participants would be counted as participants who tapered to 0 mg and did not restart, the percentages would have been 82.4% for venlafaxine, and 72.7% for paroxetine

*Intentionally left blank for privacy reasons

An increase in depressive symptoms was found in 33 out of 56 participants (58.9%) on both the quantitative and qualitative criteria; 19 out of 56 participants (33.9%) did not show an increase in depressive symptoms on either criterion. For 4 out of 56 participants (7.1%), the quantitative rating could not confirm the quantitative criteria.^2^ The agreement between the quantitative and qualitative criteria was very high (Cohen’s *κ* = 0.85).

For 10 out of 33 participants with a transition (30.3%), both quantitative and qualitative criteria indicated a sudden transition (sudden); for 14 out of 33 participants (42.4%), neither indicated a sudden transition (gradual). For 9 out of 33 participants (27.3%), quantitative criteria indicated a sudden transition, but this was not confirmed by the qualitative rating. The agreement between the quantitative and qualitative criteria was moderate (Cohen’s *κ* = 0.49). None of the participants fulfilled the qualitative criteria for a sudden transition, without fulfilling the quantitative criteria also. This indicates that the qualitative rating was more conservative in the current sample. Table 2 summarizes quantitative and qualitative criteria regarding if and how recurrence occurred.

**Table 2:** Quantitative and qualitative criteria regarding if and how recurrence occurred

Quantitative and qualitative criteria regarding *if* recurrence occurred (of *N* = 56)	Percentage (*N*)
Only quantitative criteria indicate recurrence	5.4% (*N* = 3)
Only qualitative criteria indicate recurrence	1.8% (*N* = 1)
Both quantitative and qualitative criteria indicate no meaningful change	33.9% (*N* = 19)
Both quantitative and qualitative criteria indicate recurrence	58.9% (*N* = 33)

Quantitative and qualitative criteria regarding *how* recurrence occurred (of *N* = 33)	Percentage (*N*)
Only quantitative criteria indicate sudden recurrence	27.3% (*N* = 9)
Only qualitative criteria indicate sudden recurrence	0% (*N* = 0)
Both quantitative and qualitative criteria indicate gradual recurrence	42.4% (*N* = 14)
Both quantitative and qualitative criteria indicate sudden recurrence	30.3 (*N* = 10)

**Fig. 3:**
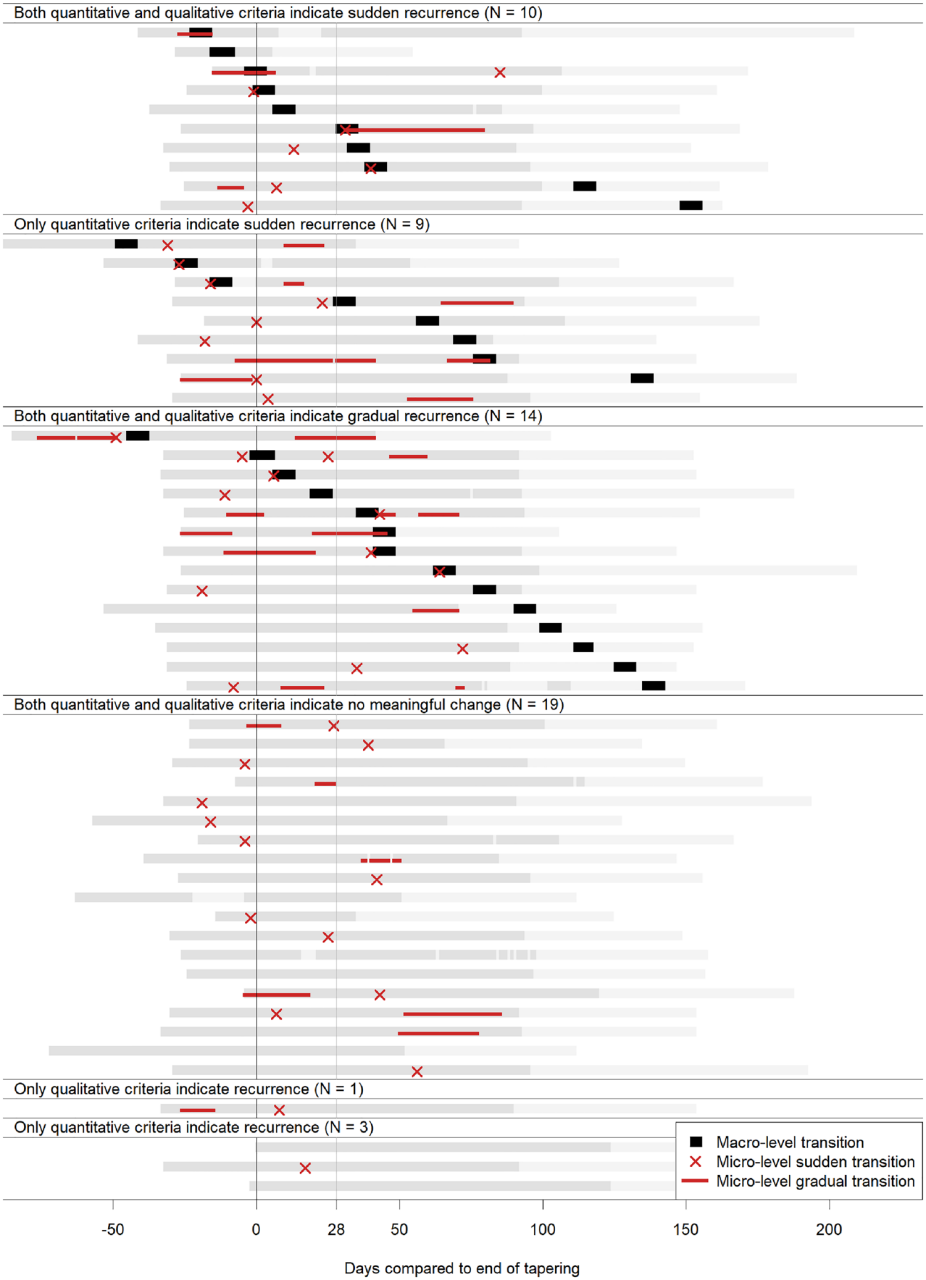
Timing of sudden and gradual transitions at the macro- and micro-level. *Note* The same black rectangles are used to indicate all three different types of macro-level transitions, (i.e., sudden, quantitative only sudden, and gradual). The heading above each section in the figure indicates which type of macro-level transition the black rectangles represent (e.g., the black rectangles under the heading “Sudden transitions (*N* = 10)” indicate sudden transitions, etc.). Gray bars in the background indicate the research period: gray indicates the part of the research period in which both macro- and micro-level assessments were obtained; light gray indicates the part of the research period in which only macro-level assessments were obtained

At the micro-level, 23 participants (41.1%) experienced only sudden increases in depressed mood, 7 (12.5%) only gradual, 17 (30.4%) experienced both types of increase, and 9 (16.1%) neither. Figure 3 shows when these increases happened in relation to the discontinuation of antidepressant medication and the timing of macro-level changes. It can be seen that in some cases, there were fewer macro-level than micro-level transitions or the other way around. In some cases, the macro-level transitions were sudden and the micro-level gradual or the other way around, and sometimes, the timing of macro and micro-level transitions did not coincide. Furthermore, both at the macro- and the micro-level, the timing of both gradual and sudden increases ranged from during antidepressant discontinuation to up to 5 months after discontinuation was completed. A substantial proportion of transitions occurred more than 28 days after antidepressant discontinuation was completed, both at the macro-level (56.3%) and at the micro-level (33.8%).

## Discussion

The current study was the first to use prospective, intensive, and multimodal data to investigate patterns regarding if, how, and when depressive symptoms reappear during and after discontinuation in individual patients. A meaningful increase in depressive symptoms was experienced by 58.9% of participants, underlining how difficult discontinuing antidepressants commonly is. Results show that both at the macro- and the micro-level of measurement, both sudden and gradual increases in depressive symptoms are common. Furthermore, all investigated types of increases in depressive symptoms or mood happened at a wide range of different timings. This shows that there is a substantial heterogeneity in the process through which depressive symptoms return, confirming the idea that different changes may be meaningful to different people.

We defined increases in depressive symptoms as ‘meaningful’ if they (a) were quantitatively large enough to be statistically confident the increase was not caused by inaccurate measurement, (b) required intervention and/or persisted for 3 weeks without intervention, and (c) the criteria above could also be confirmed in a rating of qualitative information regarding what the patient experienced in real life. Our quantitative criteria could be applied to data relatively simply and consistently as no statistical estimation was required and seemed to perform adequately as the quantitative criteria we used were confirmed by qualitative information in 92.9% of cases (Cohen’s *κ* = 0.85). By combining quantitative data at the macro-level with qualitative information, we were able to increase the likelihood that our definition of symptom transitions was valid and matched as closely as possible real and meaningful symptom changes. Though no purely quantitative method can replace the addition of qualitative assessment [40], the high agreement between the quantitative and qualitative criteria in this study suggests future researchers may consider only using weekly quantitative symptom measures or an expert rating of qualitative information. This would save time and resources, at the cost of not being able to detect the roughly 7.1% of participants for whom these criteria do not coincide. For *how* the change happened, gradually or suddenly, qualitative confirmation was found in 72.7% of cases (Cohen’s *κ* = 0.49). This suggests that both quantitative and qualitative information are important to form a complete picture of *how* meaningful change occurs. However, it is also likely that the difference between gradual and sudden increase in symptoms was harder to test quantitatively and/or rate qualitatively.

As expected, macro- and micro-level results were different, as if, how, and when an increase occurred did not seem to match for many patients. One logical explanation is that we currently used a single EMA item ‘I feel down’ to examine transitions at the micro-level, while at the macro-level symptoms such as: feeling lonely, excessive rumination, low self-esteem, and low appetite, also counted. Second, micro- an macro-level assessments are inherently different as EMA data give insight into the fleeting experience of mood in daily life while macro-level assessments provide insight into the retrospective perception of depressive symptoms of the past week. Thereby, examining change in EMA data may have added value above and beyond collecting only data at the macro-level by providing ecologically valid insight into change in emotions experienced in the daily life of patients. Related to that, we know that when people retrospectively report their experiences, their evaluation is additionally influenced by recall biases and cognitive errors [24], which do not play a major role in the micro-level data [26]. Though collecting EMA data requires a substantial investment of time and resources, it seems this cannot be replaced by weekly symptom measures. As both macro- and micro-level perspectives may yield clinically valuable information and the benefits of personalized time-series models are increasingly recognized [8, 13, 41], we expect similar designs will gain importance in this field. However, when one aims to only examine worsening of depressive symptoms over time without being interested in the experience of symptoms in daily life, using only weekly symptom measures may be an efficient solution to examine the meaningful symptom change at the within-person level.

Both at the macro- and the micro-level of measurement, both sudden and gradual increases in depressive symptoms were common. A relevant question is why transitions in depressive symptoms are gradual in some cases and sudden in others. It is sometimes assumed that gradual increases in depressive symptoms may indicate the return of depressive symptoms, whereas sudden changes may be due to discontinuation effects [42]. However, the current study shows that both gradual and sudden increases in depressive symptoms can occur months after discontinuation is completed, when the onset of discontinuation syndromes is less likely [42, 43]. Another theory that may be able to explain why symptom transitions can be gradual or sudden is ‘catastrophe theory,’ a theory originating in the field of complex dynamical systems [44, 45]. In some cases, a gradual increase in the vulnerability for depression may be directly noticeable as a gradual increase in depressive symptoms or prodromes [38]. In other cases, a similar increase in vulnerability may be difficult to notice, until vulnerability is so high that a very small perturbation in the system (e.g., an unpleasant event) is enough to trigger a sudden increase in depressive symptoms [46–49]. More research is needed to confirm this theory.

A substantial proportion (*N* = 43, 76.8%) of the sample discontinued using so-called tapering strips that allowed these participants to reduce their dose using tiny decrements [29]. It is likely that patients who have experience with failed previous attempts to taper their medication resorted to this method of tapering and thus represent a group with heightened vulnerability for unsuccessful tapering. Therefore, the high percentage of increases in depressive symptoms may not accurately generalize to the total group of people who taper their antidepressant medication. Furthermore, from the 299 individuals initially recruited, 46 declined to participate and 103 were lost to follow-up. A substantial percentage of these participants were deterred by the large number of measurements they would be required to complete when participating in this study, and the individuals that did participate may therefore represent a highly motivated subsample from the target population. Despite this potentially biased sample, the conclusion that there is a high diversity in how and when recurrence can happen remains relevant for clinical care. However, the frequency of gradual and sudden increases in depressive symptoms and the distribution of their timing may not accurately generalize to the full population of patients discontinuing antidepressant medication.

A limitation of the research design used in the current study is that it is often difficult to obtain this much within-person data, without compromising on the number of included participants. This makes it harder to perform formal between-person statistical tests. For example, the fact that participants discontinued different antidepressants in different ways may have had a relevant influence on if, how, and when increases in depressive symptoms happened, but subgroups were too small to test this statistically. This meant that due to the limited number of participants, this study was unable to test which factors and mechanisms caused the diversity in how and when depressive symptoms returned. However, the main conclusion that there is substantial diversity in how and when depressive symptoms return during and after antidepressant discontinuation does not require statistical testing at the between-person level and is therefore unaffected by this limitation. In the current study, we tried to combat this limitation as much as possible. As far as we know, the current sample size of 56 participants with a median of 27 weekly assessments of depressive symptoms, a median of 542.5 EMA self-reports, and frequent qualitative assessment was by far the largest sample of its kind to date. This allowed us to draw the important methodological conclusion that quantitative criteria based on weekly symptom measures showed a very high agreement with qualitative criteria based on what participants had experienced in daily life regarding *if* they had experienced a meaningful change, but the agreement regarding *how* this change occurred was much lower. Furthermore, we found that micro-level data regarding daily mood provided information that could not be obtained using quantitative or qualitative data at the macro-level, and EMA data may therefore be an interesting addition to studies regarding meaningful change in psychology. Using multimodal, prospective, and intensive longitudinal data, the current study presented a novel approach to investigating not only if meaningful change occurred in patients during and after antidepressant medication, but also provided insight into how and when these changes occurred.

## Data Availability

The data that support the findings of this study are available from the corresponding author upon reasonable request. The data are not publicly available due to privacy or ethical restrictions.

## Footnotes

1 For one-sided estimates, the bandwidth was adapted to have the same variance as the regular kernel: bandwidth one-sided = bandwidth/sqrt(1–2/pi)).

2 For two participants, quantitative data showed fluctuations in depressive symptoms, but participants described these fluctuations as “not at all” different from when participant was still on antidepressant medication. One participant only experienced three weeks of quantitatively elevated symptoms directly after the death of a friend and did not describe this as a return in depression. One participant did describe an increase in depressive symptoms, but criterion 1b, persistent change, was not satisfied.

## Code availability

The code that support the findings of this study is available from the corresponding author upon reasonable request.
